# Association between Intraoperative Hyperlactatemia and Myocardial Injury after Noncardiac Surgery

**DOI:** 10.3390/diagnostics11091656

**Published:** 2021-09-09

**Authors:** Jeayoun Kim, Jungchan Park, Ji-Hye Kwon, Sojin Kim, Ah Ran Oh, Jae Ni Jang, Jin-Ho Choi, Jidong Sung, Kwangmo Yang, Kyunga Kim, Joonghyun Ahn, Seung-Hwa Lee

**Affiliations:** 1 Department of Anesthesiology and Pain Medicine, Samsung Medical Center, Sungkyunkwan University School of Medicine, Seoul 06351, Korea; jeayoun.kim@samsung.com (J.K.); jc83.park@samsung.com (J.P.); jh0828.kwon@samsung.com (J.-H.K.); sojin.kim@samsung.com (S.K.); ahran.oh@samsung.com (A.R.O.); jenny.jang@samsung.com (J.N.J.); 2 Department of Medicine, Division of Cardiology, Heart Vascular Stroke Institute, Samsung Medical Center, Sungkyunkwan University School of Medicine, Seoul 06351, Korea; jin-ho.choi@samsung.com (J.-H.C.); jidong.sung@samsung.com (J.S.); 3 Department of Emergency Medicine, Samsung Medical Center, Sungkyunkwan University School of Medicine, Seoul 06351, Korea; 4 Centers for Health Promotion, Samsung Medical Center, Sungkyunkwan University School of Medicine, Seoul 06351, Korea; kmhi.yang@samsung.com; 5 Statistics and Data Center, Research Institute for Future Medicine, Samsung Medical Center, Seoul 06351, Korea; kyunga.j.kim@samsung.com (K.K.); jhguy.ahn@samsung.com (J.A.); 6 Department of Biomedical Engineering, Seoul National University College of Medicine, Seoul 06351, Korea

**Keywords:** noncardiac surgery, cardiac troponin, lactate, myocardial injury after noncardiac surgery

## Abstract

Background: Oxygen demand–supply mismatch is supposed to be one of the major causes of myocardial injuries after noncardiac surgery (MINS). Impaired tissue oxygenation during the surgery can lead to intraoperative hyperlactatemia. Therefore, we aimed to evaluate the relationship between intraoperative lactate level and MINS. Methods: A total of 1905 patients divided into groups according to intraoperative hyperlactatemia: 1444 patients (75.8%) into normal (≤2.2 mmol/L) and 461 patients (24.2%) into hyperlactatemia (>2.2 mmol/L) groups. The primary outcome was the incidence of MINS, and all-cause mortality within 30 days was compared. Results: In the crude population, the risks for MINS and 30-day mortality were higher for the hyperlactatemia group than the normal group (17.7% vs. 37.7%, odds ratio [OR]: 2.83, 95% confidence interval [CI]: 2.24–3.56, *p* < 0.001 and 0.8% vs. 4.8%, hazard ratio [HR]: 5.86, 95% CI: 2.9–12.84, *p* < 0.001, respectively). In 365 propensity score-matched pairs, intraoperative hyperlactatemia was consistently associated with MINS and 30-day mortality (21.6% vs. 31.8%, OR: 1.69, 95% CI: 1.21–1.36, *p* = 0.002 and 1.1% vs. 3.8%, HR: 3.55, 95% CI: 1.71–10.79, *p* < 0.03, respectively). Conclusion: Intraoperative lactate elevation was associated with a higher incidence of MINS and 30-day mortality.

## 1. Introduction

Myocardial injury after noncardiac surgery (MINS) is a strong independent predictor of mortality and cardiovascular morbidities [1,2,3,4,5]. The incidence of MINS is 10–25%, depending on the demographics and comorbidities of the surgical population [4]. Pre-existing cardiovascular comorbidities such as hypertension, coronary artery disease, prior myocardial infarction, heart failure, or kidney dysfunction are known to be risk factors for MINS occurrence [4]. The main mechanism is assumed to be oxygen supply–demand mismatch in more than two-thirds of MINS, but it has not been fully investigated [1].

Perioperative hyperlactatemia is a significant predictor of post-operative complications and mortality [6]. It is common, with an incidence of 37% during noncardiac surgery [7]. Low blood flow, cardiovascular comorbidities, and operative time are risk factors associated with intraoperative hyperlactatemia [7,8]. Hyperlactatemia is a marker of tissue hypoperfusion caused by oxygen supply–demand mismatch; therefore, evaluating a relationship between lactate level and MINS may provide more clues on the mechanism of MINS. [9,10]. Despite the shared pathophysiology, no scientific evidence of an association between intraoperative hyperlactatemia and MINS has been demonstrated. Therefore, we aimed to evaluate the association between intraoperative hyperlactatemia and MINS.

## 2. Materials and Methods

### 2.1. Ethical Approval

The Institutional Review Board (SMC 2019-08-048) waived the necessity to obtain informed consent for access to the Samsung Medical Center Troponin in Noncardiac Operation (SMC-TINCO) registry. SMC-TINCO is a de-identified data set that was registered at https://cris.nih.go.kr (accessed on 16 November 2020) before patient enrollment (Clinical Trial Registration: KCT0004244).

### 2.2. Study Population and Data Collection

Samsung Medical Center is a large tertiary referral center with 1989 beds that sees more than 49,000 surgical cases per year and has an electronic medical records system that includes information for more than 4 million patients. De-identified data can be extracted from this system by in-house researchers using “Clinical Data Warehouse Darwin-C”. The SMC-TINCO registry was generated using this system and contains a de-identified data set of 43,019 patients who had at least one cTn I measurement preoperatively or within one month after noncardiac surgery at Samsung Medical Center from January 2010 to June 2019.

From the SMC-TINCO registry, we selected adult patients with postoperative cTn I and intraoperative lactate measurements. The enrolled patients were divided into two groups according to intraoperative lactate level: normal and intraoperative hyperlactatemia (>2.2 mmol/L) groups. For baseline characteristics, independent investigators were blinded to the cTn I level, reviewed the preoperative evaluation, and organized it into a standardized form.

### 2.3. Study Outcome and Definitions

The primary study outcome was the incidence of MINS, defined as a postoperative cTn elevation greater than the 99th percentile upper reference limit due to cardiac ischemia within 30 days after surgery [1]. Patients who experienced cTn elevation from a non-ischemic cause such as tachyarrhythmia, defibrillation or cardioversion, pulmonary thromboembolism, chronic troponin elevation, or sepsis were not regarded as having MINS [1]. The secondary outcome was mortality during the 30 days after surgery. Mortality statistics were updated according to the National Population Registry of the Korea National Statistical Office.

Ischemic heart disease was defined as a history of medication or intervention for coronary artery disease. We defined heart failure as a history of heart failure, or the use of loop diuretics accompanied by symptoms including diastolic heart failure with preserved left ventricular systolic function. Arrhythmia was defined as a history of atrial fibrillation, atrioventricular block, paroxysmal supraventricular tachycardia, or unspecified arrhythmia. We determined the risk of surgery according to the European Society of Cardiology and the European Society of Anesthesiology guidelines in 2014 [11]. Active cancer was defined when the histologic diagnosis of cancer occurred within six months of the study period.

### 2.4. Perioperative cTn I Measurement

At our institution, perioperative cTn measurement was recommended for patients scheduled for moderate- to high-risk surgery and those with more than one of the risk factors in the revised cardiac risk index based on the current guidelines [11,12]. For patients who did not fulfill the criteria, cTn was measured at the discretion of the attending clinician based on past medical history or suspected ischemic symptoms. An automated analyzer with Tn I-Ultra immunoassay (Advia Centaur XP, Siemens Healthcare Diagnostics, Erlangen, Germany) was used. The 99th percentile upper reference limit was 40 ng/L, and the lower detection limit was 6 ng/L according to the manufacturer.

### 2.5. Intraoperative Lactate Measurement

To identify hyperlactatemia, the cutoff value was 2.2 mmol/L, according to the upper normal limit for our institutional laboratory. Intraoperative lactate level was measured by a blood gas analyzer (RAPIDLab 1200 Blood Gas Analyzer; Siemens Healthcare, Erlangen, Germany) at 37 °C and was obtained from the pre-placed arterial line using heparinized blood gas syringes. The arterial line was placed mostly for real-time blood pressure monitoring in cases where pharmacologic or mechanical cardiovascular manipulation was anticipated, more than 500 mL of blood loss was predicted, or repeated blood sampling was needed. Generally, blood samplings were taken after anesthetic induction for the baseline values and at a 2 h time interval during the surgery. Additional samplings were performed at the discretion of the attending anesthesiologist.

### 2.6. Statistical Analysis

The categorical variables are presented as numbers and percentages (%), and the two groups were compared with Fisher’s exact tests or chi-squared tests. Continuous variables that were normally distributed are presented as the mean ± standard deviation, and variables with skewed distribution are presented as the median and interquartile range (IQR). Comparisons of two groups were conducted using the Student’s *t*-test or the Mann–Whitney U test, as applicable. To compare MINS occurrence between groups, rigorous adjustments were conducted using a multivariable logistic regression model, and the results are reported as the adjusted odds ratio (OR) with a 95% confidence interval (CI). We compared 30-day mortality using Cox proportional-hazard regression analysis and reported it as the hazard ratio (HR) and 95% CI. Propensity score matching analysis was conducted to maximize study power and reduce selection bias by balancing variables between the two groups. Absolute standard mean deviation (ASD) values under 10% indicated successful propensity score matching with an appropriate balance between the groups. In the matched population, a logistic regression model was used to compare the OR for MINS, and the Cox regression model was used to compare HR for 30-day mortality. We used Pearson’s correlation coefficients and receiver operating characteristic (ROC) curves to determine the optimal threshold of cTn I to predict MINS, and the sensitivity and specificity of the threshold were calculated.

The statistical power of the study considering sample size was assessed by Spearman’s rank correlation [13]. The study power was 0.8 when the OR was 1.5, and 0.93 when the OR was 1.65. For sensitivity analysis, we estimated the potential impact of unmeasured confounders. We assumed that unmeasured confounders had an incidence of 40% and estimated the changes in HR and CI according to the associations of unmeasured confounders with MINS and intraoperative hyperlactatemia [14]. We performed all statistical analyses using R (version 4.0.2, R Foundation for Statistical Computing, Vienna, Austria).

## 3. Results

### 3.1. Patient Characteristics

**Figure 1:**
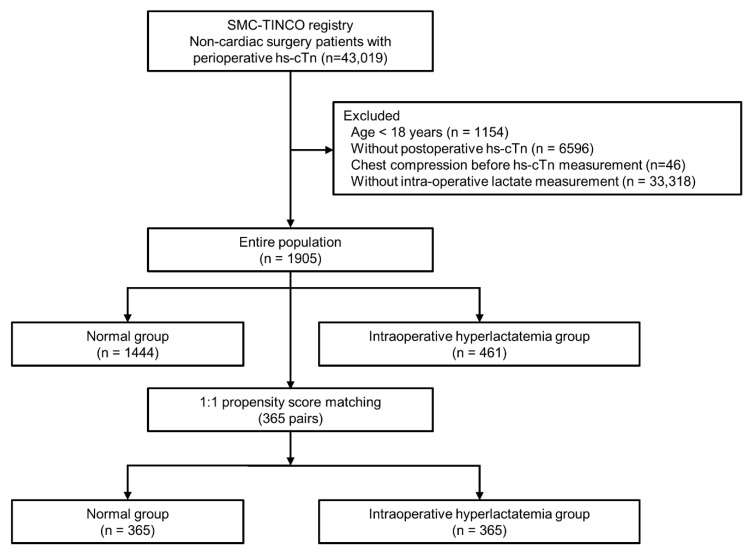
The flow chart of patients. SMC-TINCO: Samsung Medical Centre Troponin in Noncardiac Operation.

Among the total 43,019 patients in the SMC-TINCO registry, 1154 patients younger than 18 years old, 46 patients who received chest compressions before cTn measurement, and 6596 patients without postoperative cTn measurement were excluded. Finally, 1905 patients with at least one intraoperative lactate measurement were included in the analysis. These patients with intraoperative plasma lactate measurements were divided into two groups, normal and intraoperative hyperlactatemia groups. A total of 1444 patients (75.8%) had normal intraoperative lactate levels, and 461 patients (24.2%) showed intraoperative hyperlactatemia (Figure 1). Baseline characteristics and perioperative variables are presented in Table 1. Although the hyperlactatemia group was younger, these patients were more likely to have comorbidities such as smoking and diabetes than the normal group. Preoperatively, the hyperlactatemia group more frequently received intensive care, including continuous renal replacement therapy and ventilator support. Among operative variables, operation time tended to be longer in the hyperlactatemia group, and emergency and high-risk surgery according to ESC/ESA guidelines, red blood cell transfusion, and the continuous infusion of intraoperative inotropic drugs were more frequent in the hyperlactatemia group. Surgery types are summarized in Table S1. After propensity score matching, a well-balanced data set of 365 pairs was generated with ASD less than 10 (Table 1).

### 3.2. Clinical Outcomes

Postoperative cTn I was elevated in 23.6% (450/1905) of the crude population. Among these patients, 21 were not regarded as suffering from MINS, because cTn I was elevated due to non-ischemic etiology. The incidence of MINS in the entire population was 22.5% (429/1905). The median duration from end of a surgery to the peak postoperative cTn I was 0.53 (IQR: 0.08–1.6) days in the normal group and 0.3 (IQR: 0.06–1.53) days in the intraoperative hyperlactatemia group. After adjustments with multivariable analysis, the risks of MINS and 30-day mortality were higher in the patients with intraoperative hyperlactatemia (37.7% vs. 17.7%, OR: 2.03, 95% CI: 1.53–2.69, *p* < 0.001 and 4.8% vs. 0.8%, HR: 2.61, 95% CI: 1.13–6.03, *p* = 0.02, respectively; Table 2).

Consistent results were shown in propensity-score-matched populations. The incidence of MINS was higher in the hyperlactatemia group than the normal group (31.8% vs. 21.6%, OR: 1.69, 95% CI: 1.21–2.36, *p* = 0.002; Table 2), as was 30-day mortality (3.8% vs. 1.1%, HR: 3.55, 95% CI: 1.17–10.79, *p* = 0.03; Table 2). Sensitivity analysis was conducted to evaluate the effects of unmeasured confounders on the observed association. Under all circumstances, the statistical significance of the association was maintained (Table S2).

### 3.3. Threshold

**Figure 2:**
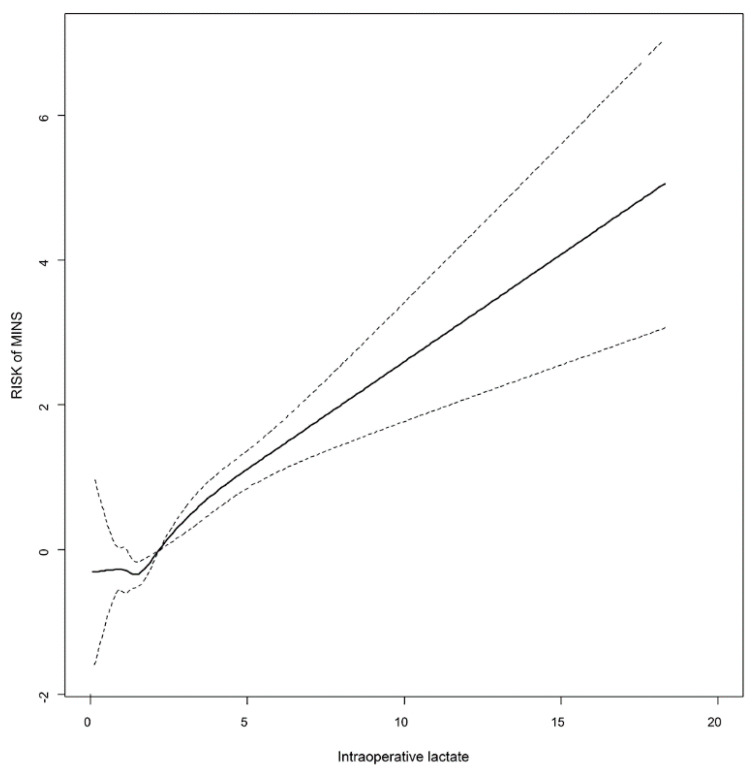
Estimated risk (95% confidence interval) of myocardial injury after noncardiac surgery according to lactate concentrations in the entire population. The dotted line represents confidence interval.

**Figure 3:**
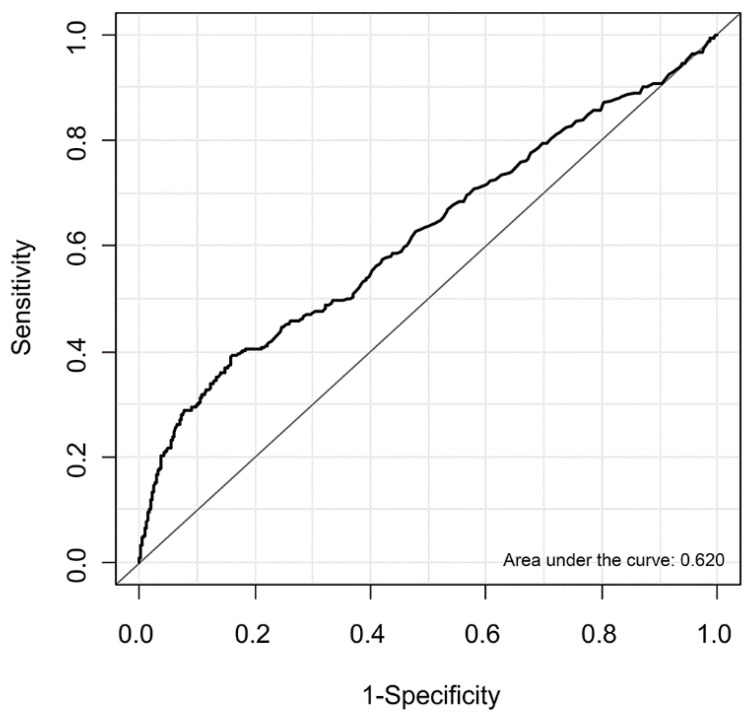
Receiver operating characteristic curves of the entire population for intraoperative lactate concentration associated with myocardial injury after noncardiac surgery.

Changes in the risk of MINS of the entire population according to lactate concentration were estimated and are shown in Figure 2. The optimal threshold for intraoperative lactate level to predict MINS was 2.38 mmol/L according to the ROC analysis. The area under the ROC curve was 0.62. Based on this value, sensitivity and specificity were 39.2% and 83.9%, respectively (Figure 3).

## 4. Discussion

The main finding of this study was that elevated intraoperative lactate levels were significantly associated with the occurrence of MINS (OR: 1.69) and 30-day mortality (HR: 3.55). These findings support the link between oxygen demand–supply mismatch and MINS.

Hyperlactatemia is well known for its association with poor outcomes in various populations [15,16,17,18,19,20,21]. Similarly, we exhibited a higher risk of MINS and 30-day mortality in patients with intraoperative hyperlactatemia. In broad terms, elevated lactate can be divided into two categories: cases where it is driven by impaired tissue oxygenation (type A) and cases where tissue oxygenation is maintained (type B) [22]. Previous studies explained intraoperative hyperlactatemia mainly with oxygen supply–demand mismatch caused by tissue hypoperfusion [7,8]. The mismatch is one of the main mechanisms of MINS [1,2,3]. We assumed that the intraoperative pathologic status causing a lactate elevation could also cause oxygen supply-demand mismatch of the myocardium and lead to the higher incidence of MINS. In our study, the hyperlactatemia group had a higher prevalence of diabetes and smoking and the need for intensive care. In addition, the proportions of emergency and high-risk surgery were higher in the hyperlactatemia group. We suppose that worse baseline and operative characteristics made them more susceptible to tissue hypoperfusion and hypoxia [7,8]. Greater needs for blood transfusion and inotropics during the surgery supported the intraoperative hypoperfusion state of the hyperlactatemia group. Operative time is another risk factor for hyperlactatemia. Prolonged surgical trauma may cause local acidosis and lead to microcirculatory alterations, causing an imbalance between oxygen delivery and consumption [7,8,23].

However, the predictive power of lactate for the occurrence of MINS was slightly low (AUC = 0.62). We attributed this to multifactorial causes of intraoperative hyperlactatemia. Perioperative variables may induce lactate elevation without tissue hypoxia (type B). For example, metformin, commonly prescribed for diabetic patients, accelerates lactate production and reduces lactate metabolism [24]. Sepsis or the epinephrine-induced stimulation of B2-adrenoreceptor augments lactate production without tissue hypoxia [22]. Regarding the contribution of tissue hypoxia in intraoperative hyperlactatemia, further studies are needed.

The association of intraoperative hyperlactatemia with MINS could provide a deeper understanding of the pathophysiology underlying MINS. MINS is an independent predictor of mortality and cardiovascular morbidities, but the relevant pathophysiology and modifiable risk factors have not been fully investigated [1,2,3]. For myocardial infarction, the main pathophysiology in the perioperative period was more likely to be supply–demand mismatch than the thrombus [1,25,26,27,28,29]. However, previous studies were small-sized and did not include patients with isolated cTn elevation without ischemic symptoms. Therefore, the role of oxygen demand–supply mismatch in MINS remains uncertain. In this study, patients with intraoperative hyperlactatemia showed a significantly high incidence of MINS even after we adjusted for confounding factors. These findings support the link between oxygen demand–supply mismatch and MINS.

Our study adds the following to the previous literature. First, we assessed patients with intraoperative hyperlactatemia during noncardiac surgery. Previous studies have been conducted mainly in cardiac surgery [15,16,30] or the setting of critical illness [17,18,19,20,21]. Additionally, they only measured postoperative lactate levels. Thus, the influence of the intraoperative status could not be assessed. Several studies have evaluated mortality or other postoperative complications associated with elevated lactate levels in various surgeries [30,31,32,33,34,35,36], but the association has never been evaluated specifically between intraoperative lactate levels and MINS, which share a common mechanism-related oxygen demand and supply mismatch. We demonstrated that patients with intraoperative hyperlactatemia had an increased risk of MINS. For clinical implications, the advantage of lactate is that it is a readily available parameter in the operative setting and can detect occult hypoperfusion [34]. An elevated lactate level may alert clinicians to reassess the patient and correct modifiable factors. Increasing the oxygen supply while maintaining optimal cardiac output, blood pressure, heart rate, arterial oxygen gas pressure, and hemoglobin is known to help reduce lactate levels [37,38].

Our study has several limitations. First, this was a retrospective and observational study. Although we adjusted for various confounding factors using multivariable and propensity-score-matched analysis, unmeasured confounding factors and selection bias might have persisted. Even after we adjusted for confounding factors, more patients in the hyperlactatemia group received preoperative intensive care and continuous renal replacement therapy. Worse preoperative conditions could have a higher incidence of MINS and 30-day mortality. A prospective, matched study could bring reliable outcomes for the primary question. Second, the cause of intraoperative hyperlactatemia can be multifactorial. Third, we did not consider the effect of types of surgery. Instead, we adjusted the risk of surgery considering the proportion of high-risk surgery as a confounding factor. Additionally, management strategies for intraoperative hyperlactatemia differed among attending clinicians in terms of optimizing cardiac output, blood pressure, treating arrhythmia, transfusion, and oxygen supply. This variation might have affected the incidence of MINS. Finally, we are unable to suggest any measures to decrease hyperlactatemia or MINS.

Despite these limitations, our study demonstrated an association between intraoperative hyperlactatemia and MINS, providing a clue to the pathophysiology of MINS. Our findings are valuable to explore future randomized trials of strategies for the prevention and treatment of MINS.

## 5. Conclusions

Intraoperative hyperlactatemia was associated with a higher incidence of MINS. Further studies are needed to confirm this.

## Figures and Tables

**Table 1:** Baseline characteristics of the entire population according to intraoperative hyperlactatemia.

	Entire Population			Propensity Score Matched Population		
	Normal (*N* = 1444)	Hyperlactatemia (*N* = 461)	*p* Value	Normal (*N* = 365)	Hyperlactatemia (*N* = 365)	SMD
Peak cardiac troponin level, ng/L	910 (±15,508)	650 (±3646)	0.72	327 (±2642)	552 (±3177)	7.7
* Days to peak cardiac troponin	0.53 (0.08–1.60)	0.3 (0.06–1.53)	0.13	0.20 (0.06–0.94)	0.39 (0.07–1.71)	10.2
Male	903 (62.5)	297 (64.4)	0.5	236 (64.7)	234 (64.1)	1.1
Age	63.7 (±12.8)	60.4 (±13.1)	<0.001	61.6 (±13.5)	61.9 (±13.3)	1.7
Diabetes	846 (58.6)	362 (78.5)	<0.001	260 (71.2)	275 (75.3)	9.3
Hypertension	904 (62.6)	270 (58.6)	0.14	230 (63.0)	219 (60.0)	6.2
Current smoking	102 (7.1)	52 (11.3)	0.01	34 (9.3)	40 (11.0)	5.4
Current alcohol	231 (16.0)	91 (19.7)	0.07	74 (20.3)	77 (21.1)	2
Chronic kidney disease	143 (9.9)	45 (9.8)	1	33 (9.0)	33 (9.0)	<0.1
Active cancer	761 (52.7)	198 (43.0)	<0.001	174 (47.7)	178 (48.8)	2.2
*Previous disease*						
History of ischemic heart disease	257 (17.8)	64 (13.9)	0.06	53 (14.5)	55 (15.1)	1.5
History of heart failure	23 (1.6)	7 (1.5)	1	6 (1.6)	6 (1.6)	<0.1
History of stroke	111 (7.7)	30 (6.5)	0.46	28 (7.7)	26 (7.1)	2.1
History of arrhythmia	108 (7.5)	30 (6.5)	0.55	24 (6.6)	23 (6.3)	1.1
History of heart valve disease	19 (1.3)	3 (0.7)	0.36	5 (1.4)	3 (0.8)	5.3
*Preoperative care*						
* Intensive care unit	31 (2.1)	58 (12.6)	<0.001	16 (4.4)	32 (8.8)	17.8
ECMO	0 (0.0)	0 (0.0)	>0.99	0 (0.0)	0 (0.0)	<0.1
* Continuous renal replacement therapy	2 (0.1)	19 (4.1)	<0.001	2 (0.5)	10 (2.7)	17.3
Ventilator	5 (0.3)	11 (2.4)	<0.001	4 (1.1)	2 (0.5)	6.1
*Operative variables*						
ESC/ESA surgical high risk	394 (27.3)	286 (62.0)	<0.001	183 (50.1)	191 (52.3)	4.4
Emergency operation	139 (9.6)	117 (25.4)	<0.001	80 (21.9)	84 (23.0)	2.6
General anesthesia	1439 (99.7)	455 (98.7)	0.045	362 (99.2)	359 (98.4)	7.5
Operation duration, hours	3.53 (±1.88)	5.02 (±2.58)	<0.001	4.33 (±2.38)	4.55 (±2.44)	9.2
Continuous infusion of inotropics	419 (29.0)	267 (57.9)	<0.001	181 (49.6)	184 (50.4)	1.6
Intraoperative RBC transfusion	422 (29.2)	224 (48.6)	<0.001	136 (37.3)	149 (40.8)	7.3

Data are presented as n (%), mean (±standard deviation) or median (interquartile range). SMD, standardized mean difference; ECMO, extracorporeal membranous oxygenation; ESC, European Society of Cardiology; ESA, European Society of Anaesthesiology; RBC, red blood cell. * These variables are not retained in propensity score matching.

**Table 2:** The incidence of myocardial injury after noncardiac surgery according to intraoperative hyperlactatemia.

	Normal	Hyperlactatemia	Unadjusted OR/HR (95% CI)	*p* Value	* Adjusted OR/HR (95% CI)	*p* Value
Entire population	(*N* = 1444)	(*N* = 461)				
MINS	255 (17.7)	174 (37.7)	2.83 (2.24–3.56)	<0.001	2.03 (1.53–2.69)	<0.001
30-day mortality	12 (0.8)	22 (4.8)	5.86 (2.90–11.84)	<0.001	2.61 (1.13–6.03)	0.02
Propensity-score-matched population	(*N* = 365)	(*N* = 365)				
MINS	79 (21.6)	116 (31.8)			1.69 (1.21–2.36)	0.002
30-day mortality	4 (1.1)	14 (3.8)			3.55 (1.17–10.79)	0.03

Data are presented as n (%). MINS is presented with OR, and mortalities are presented as HR. * Multivariable analysis included age, operation duration, diabetes, smoking, coronary artery disease, peak cardiac troponin level, ESC/ESA surgical high risk, general anesthesia, emergency operation, active cancer, continuous infusion of inotropics, and intraoperative RBC transfusion. Abbreviations: MINS, myocardial injury after noncardiac surgery; OR, odds ratio; HR, hazard ratio; CI, confidence interval; ESC, European Society of Cardiology; ESA, European Society of Anaesthesiology; RBC, red blood cell.

## Data Availability

The data are not publicly available due to our institutional guidelines.

## Supplementary Materials

The following are available online at https://www.mdpi.com/article/10.3390/diagnostics11091656/s1, Table S1: Types of Surgery. Table S2: Effect of an unmeasured confounder on the odds ratio of intraoperative hyperlactatemia on myocardial injury after noncardiac surgery in propensity-score-matched population.
