# Supplementary material for: Association between Intraoperative Hyperlactatemia and Myocardial Injury after Noncardiac Surgery
Source: Diagnostics (Basel). 2021 Sep 9;11(9):1656. doi: 10.3390/diagnostics11091656 (PMC8465750; doi:10.3390/diagnostics11091656)
Supplement: Supplementary file 1 [file diagnostics-11-01656-s001.zip › diagnostics-1358916-supplementary.pdf]

Table S1. Types of surgery.

	**Normal (*N* = 1444)**	**hyperlactatemia (*N* = 461)**
Vascular	121 (8.4)	49 (10.6)
Orthopediatric	62 (4.3)	4 (0.9)
Neuro	165 (11.4)	71 (15.4)
Breast or Endo	4 (0.3)	2 (0.4)
Plastic or Otolaryngeal or Eye	25 (1.7)	8 (1.7)
Transplantation	104 (7.2)	125 (27.1)
Gynecology or Urology	128 (8.9)	20 (4.3)
Gastrointestinal	418 (28.9)	161 (34.9)
Noncardiac thoracic	416 (28.8)	20 (4.3)
Others	1 (0.1)	1 (0.2)

Table S2. Effect of an unmeasured confounder on odds ratio of intraoperative hyperlactatemia on myocardial injury after noncardiac surgery in propensity-score matched population.

		**$OR_{ZY\|X}$**					
		**1.5**	**2**	**2.5**	**3**	**3.5**	**4**
	0.3	2.04 (1.43-2.91)	2.31 (1.61-3.33)	2.54 (1.75-3.68)	2.78 (1.90-4.07)	3.06 (2.07-4.52)	3.23 (2.17-4.81)
	0.4	1.93 (1.36-2.73)	2.09 (1.47-2.97)	2.28 (1.59-3.26)	2.41 (1.67-3.47)	2.55 (1.76-3.70)	2.72 (1.86-3.97)
OR_{zx}	0.5	1.85 (1.32-2.61)	1.99 (1.41-2.82)	2.10 (1.47-2.98)	2.20 (1.54-3.15)	2.30 (1.60-3.31)	2.42 (1.67-3.50)
	0.6	1.80 (1.28-2.53)	1.89 (1.34-2.66)	1.98 (1.40-2.80)	2.07 (1.46-2.94)	2.14 (1.50-3.06)	2.21 (1.54-3.18)
	0.7	1.77 (1.26-2.47)	1.83 (1.31-2.58)	1.89 (1.34-2.67)	1.96 (1.38-2.77)	2.01 (1.41-2.86)	2.06 (1.44-2.94)

Prevalence of unmeasured confounder = 40%.

Numbers represent ORs (including 95% CIs).

OR, odds ratio; X, dichotomous exposure measure; y, dichotomous outcome measure; z, potential dichotomous confounder.

ORZX indicates the association (OR) between the unmeasured confounder and intraoperative hyperlactatemia.

ORZY|X indicates the association (OR) between the unmeasured confounder and myocardial injury after noncardiac surgery.
